# Aesthetics for everyday quality: one way to enrich healthcare improvement debates

**DOI:** 10.1136/medhum-2021-012330

**Published:** 2022-02-24

**Authors:** Alan Cribb, Graham Pullin

**Affiliations:** 1 Centre for Public Policy Research, King's College London, London, UK; 2 Duncan of Jordanstone College of Art and Design, University of Dundee, Dundee, UK

**Keywords:** arts in health/arts and health, Medical humanities, philosophy of medicine/health care, health care education

## Abstract

In this paper we seek to illuminate the importance of aesthetics for healthcare quality and encourage more explicit discussion of aesthetics in healthcare improvement scholarship and practice. We hope to contribute to and help develop the hinterland between arts-based initiatives in healthcare and the ‘normal business’ of healthcare quality improvement. Our broad contention is: (1) That aesthetic considerations should be seen as of universal relevance across quality debates (2) That they never be assumed to have a marginal or even secondary status; and (3) That taking aesthetic considerations seriously calls for explicit discussion of associated uncertainties and dilemmas and a readiness to welcome aesthetics expertise into improvement debates.

In this paper we aim to illuminate the importance of aesthetics for healthcare quality and encourage more discussion of aesthetics in healthcare improvement scholarship and practice. We will not be focussing directly on arts-based initiatives in health—although we see that as a hugely important topic, clearly linked to our concerns, and one we will touch on in a few places. Here we are more interested in what has been called ‘everyday aesthetics’ (Light and Smith 2005; Saito 2007) and its relevance for understanding and pursuing what might be called ‘everyday quality’. Our argument is that there is much to be gained by making debate about aesthetics a more routine and pervasive part of healthcare quality improvement.

We hope to explore and contribute to the hinterland between arts-based initiatives in healthcare and the ‘normal business’ of healthcare quality improvement. The idea of quality improvement can be interpreted in an expansive way or in a more restricted way. The former accommodates the full range of means through which, and respects in which, healthcare can be made better. The latter typically refers to a specialist domain now incorporated within healthcare policy and organisations—which we will label with the capitalised ‘QI’—in which professionals with specific improvement-related expertise set out to systematically monitor and strengthen indicators of quality. Arts-based work unquestionably has a huge contribution to make to quality improvement in the broader sense and can also provide important complementary insights and tools for QI more narrowly understood (Gardner *et al.* 2021). But the relevance of aesthetics extends beyond the arts. This is something perfectly familiar from routine experience outside healthcare. People take aesthetic considerations into account, so far as they are in a position to do so, in their decisions about where to live, where to spend their leisure time, what to eat and drink, and so on. Aesthetics is also something recognised within healthcare QI discourses although this emphasis is often confined to specific areas of concern, such as, for example, healthcare architecture.

The idea of ‘aesthetics’ is the subject of a fundamental, wide-ranging and complex set of philosophical debates. For example, there are debates about how far aesthetic qualities should be seen as belonging to ‘objects’ such as paintings or landscapes (and so on) or to the experiences or judgements of people encountering such ‘objects’. There are disagreements about whether, or the senses in which, aesthetic judgements should be seen as ‘objective’ or ‘subjective’. And there are rival candidates for defining the subject matter of aesthetics (Budd 2008). Obviously, we will not be trying to resolve these foundational debates here. For our purposes we can largely leave such philosophical questions open. But we will be suggesting that there are a number of important practice-facing questions, with roots in these fundamental debates, that deserve attention within healthcare improvement.

The approach we adopt in this paper will draw on Yuriko Saito’s account of ‘everyday aesthetics’ (2007) and we will expand on this construction of the aesthetic shortly. However, the account we employ will, in large measure, overlap with a quite familiar ‘common sense’ usage of the term. As already indicated no one is a stranger to aesthetics and we make aesthetic judgements on a regular basis without the idea seeming mysterious to us. We can admire someone’s coat or we can feel dismayed by the massively cluttered state of a room, and we can do so without feeling the need for an account of the nature of aesthetics. These examples are enough to indicate that aesthetic considerations represent an additional dimension to both technical and ethical considerations (although, as we will go on to discuss, these different dimensions can inform one another in significant ways). To admire the look, style and feel of a coat is not to say that it is a ‘good coat’ in other respects—it may not, for example, be particularly weather-resistant or made of ecofriendly materials. Similarly, if we have a cluttered room this may make it a poor room for us to occupy and make use of, and we may also feel it represents a kind of ethical failure on our part but, in addition, we can regard it as aesthetically unsatisfactory.

This familiar usage also occurs in the QI literature. For example, in Donabedian (1989) classic account of healthcare quality he identifies three quality components ‘the goodness of technical care, judged by its effectiveness, the goodness of the interpersonal relationship, judged partly by its contribution to technical care, and the goodness of the amenities’ (p3); going on to expand on the last component as follows: ‘the goodness of the amenities of care, by which I mean convenience, creature comforts, and even the aesthetic attributes of the setting in which care is provided’ (p4). A similar, but much more recent, example is found in Slater *et al*’s Person-centred Practice Inventory (2017) that sets out to operationalise person-centred practice as ‘an internationally recognised standard of quality care’ by using 17 constructs (informed by a Delphi study of experts) including one summarised as: *‘The physical environment*: Healthcare environments that balance aesthetics with function by paying attention to design, dignity, privacy, sanctuary, choice/control, safety and universal access with the intention of improving patient, family and staff operational performance and outcomes’ (p544). Here aesthetics is invoked in relation to person-centredness and healthcare environments and we will return to both these ideas and the links between them as we proceed.

For the most part mainstream QI literature does not feature prominent discussion of aesthetics. An important exception is that part of the literature that makes use of design thinking where the relevance of aesthetics is accepted as standard (Parsons 2016). For example, work on Experience Based Co-Design (which we come back to in the final section) embraces aesthetics as a key concern (Bate and Robert 2007). Nonetheless there is a clear tendency in the QI literature for aesthetics to fall into the background and, in particular, for overt discussion of aesthetics to be marginal. There is perhaps something ‘off-putting’ or alienating about the term ‘aesthetics’, but leaving this thought aside for now, some of the reason for the relative marginality might be indicated by the examples cited above. In particular the QI examples mentioned suggest that aesthetic considerations are first, related to a relatively circumscribed portion of healthcare quality evaluation and second, form a relatively low priority compared with technical and ethical considerations. It is notable that Donabedian, while mapping out what he sees as the essence of quality, includes aesthetics using the phrase ‘*even* the aesthetic attributes’ (our emphasis). In the remainder of this paper we will push against these associations. We will suggest: (1) That aesthetic considerations should be seen as of universal relevance across quality debates (just as technical and ethical considerations are accepted as being); (2) That they should never be assumed to have a marginal or even secondary status; and (3) That taking aesthetic considerations seriously entails some explicit discussion of associated uncertainties and dilemmas and a readiness to welcome aesthetics expertise into improvement scholarship.

## Everyday aesthetics: key ideas and implications

Here we are drawing on the growth of scholarship on the aesthetics of everyday life (eg, Light and Smith 2005; Naukkarinen 2017), and in particular to Saito’s two books on the subject (Saito 2007,^2017^). This contemporary scholarship builds on earlier contributions, including notably that of Dewey (1958), that separate out ‘aesthetic experiences’ from art. Saito’s account of everyday aesthetics builds on, but is not reducible to, work in environmental aesthetics, Japanese aesthetics and feminist aesthetics. It seeks to uncover and explore the importance of aesthetic considerations beyond the sphere of art (obviously without denying the important place of the arts in the field of aesthetics.) This involves two key moves. First, it involves not seeing aesthetics as primarily ‘art-centred’ such that things only become of interest to aesthetics purely for the reason that, and to the extent that, they are either art or proximate to art. For example, we do not need to be able to ‘translate’ our aesthetic appreciation of some aspect of nature into its ‘art-like’ qualities and status for it to be valid. Second, it involves not limiting the category of the aesthetic to a subset of ‘special’ experiences that fall outside the normal ‘flow of life’. Clearly this does not involve denying that some aesthetic experiences can somehow stand out from, ‘cut-across’, transcend or transform life in profound ways. It is rather simply asserting that not all experiences need to fall into this set to qualify as aesthetic.

These two moves have very substantial implications. They help enable us to see how aesthetic concerns need not be ‘distanced’ from routine concerns but are deeply embedded in all aspects of our lives. The risk, otherwise, is that the ‘assumptive worlds’ we occupy place aesthetic considerations either ‘outside’ ordinary life or, when they are acknowledged as ‘inside’ it, position them as thereby relatively inconsequential. (A rough comparator here might be ‘play’—if we see ‘play’ as bracketed off from normal life and chiefly only having significance in its own terms it is tempting to see it as being unimportant ‘in real life’.) These two moves encourage us to approach and think about the way aesthetics, in the context of our day-to-day lives, is entangled with a range of practical and substantive agendas and activities. We will illustrate some of these questions shortly but before that we should sketch in the notion of the ‘aesthetic’ that Saito operates with (drawing on the work of other thinkers including Carroll (2001)).

Her summary and very broad account of the aesthetic is ‘any reactions we form towards the sensuous and/or design qualities of any object, phenomenon or activity’ (2007, p9). We can fill this in a little further, showing how it is continuous with but also ‘stretches’ the common-sense use of the aesthetic mentioned above. In this effort it is still worth keeping in mind some of the well-established associations of aesthetics with the arts, because at the same time as rejecting the idea that aesthetics are to be wholly equated with, or defined in terms of, the arts, we can still see the arts as a powerful set of analogues for extra-artistic aesthetic concerns. Our reactions to ‘sensuous’ qualities obviously include our attraction to or aversion from the ‘appearance’ of ‘objects’ (here used as shorthand to also encompass the ‘phenomena’ and ‘activities’ mentioned in the summary account)—crudely the delight, displeasure or even disgust we take in the way things look. But, of course, these basic ideas are equally applicable to the way things sound, feel, smell or taste too. For Saito this already indicates one potential gap between art-centred aesthetics and everyday aesthetics because the former has traditionally been centred more around some senses rather than others. An interest in everyday aesthetics, as well as extending beyond the appearance of objects, includes the possibility of noting and considering the beauty manifested by 'everyday things': mundane objects, including such things as domestic utensils, that might not usually be thought worthy of such consideration (Yanagi 1926).

But the kinds of appreciation, satisfaction or fulfilment we can take in experiencing or actively engaging with ‘objects’ extend well beyond their immediate sensory qualities. When we engage with a garden, for example, whether as a spectator or a gardener, our appreciation can also relate to its overall composition and form, how it relates to our own and others’ identities and biographies, the garden’s recent and/or longer-term history and the allusions made or conjured up by it, the ambience it sits within and produces, and so on. In short in aesthetic appreciation ‘sensuous qualities’ are mixed with, and shaped by, many other qualities including those relating to the ‘forms’, ‘meanings’ and ‘purposes’ that objects embody. This is well understood in the arts where we would never imagine that the aesthetic significance of a novel or a musical piece was somehow wholly contained by our direct sensory experience of a particular series of words or sounds. In the summary account above Saito uses the shorthand ‘design’ to point towards these broader features of objects that includes their composition and significance which also inform our aesthetic appreciation of them as, for example, harmonious, elegant or uplifting. The language of ‘design’ of course brings into view all kinds of artefacts, spaces, events or organisational processes that are consciously designed but it is here being used in a much more extensive, including metaphorical, sense in which ‘design’ need not imply a designer. This is because one of the contrasts that Saito wants to make between art-centred aesthetics and the aesthetics of everyday life is that the latter does not require a clearly demarcated object produced by an artist or team of artists or equivalent. One of the examples she offers is the way that we can have complex aesthetic reactions to visiting cities like New York which arise from constellations of interacting experiences—ordered and disordered—that have not been deliberately or coherently assembled or curated and which have no clear boundaries.

To conclude this very brief summary of everyday aesthetics it is worth highlighting a few key points that begin to indicate the potential implications for healthcare quality. Most important, once we recognise the way aesthetic concerns are routinely entangled in everyday life, experience and activity (and do not only ‘sit outside’ ordinary life) then it follows that in order to understand and influence our practical affairs we need to ensure the aesthetic dimension is included in the frameworks that we use to understand and change things. Aesthetic reactions inform the choices we make and the preferences and priorities we have in both our private and public lives and this happens without us labelling them as such or even necessarily noticing. Nor is there any reason to think of these aesthetic considerations as being confined to relatively superficial or trivial matters. One area that clearly illustrates this—where the interface between the aesthetic and the practically urgent is something people are increasingly conscious of—is the impact of our aesthetic choices on the environment. As Saito puts it, ‘seemingly insignificant everyday preferences and decisions can have serious environmental, moral, social, political and existential implications’ (2007, p53). The example of rising environmental consciousness can also serve as a reminder of a couple of other insights about everyday aesthetics. First, as already indicated, aesthetic reactions can be negative as well as positive—we can, for example, have aversive aesthetic reactions to polluted beaches and waters. Second, while people’s aesthetic reactions can serve an important explanatory function we do not need to treat them as fixed. Rather it is possible for them to be socially and culturally influenced and shaped in various ways—this is illustrated by the way that both commercial branding and many people’s habitual tastes have evolved in the light of the green movement.

In the following two sections we will use a few examples both to illustrate the central relevance of aesthetics to healthcare quality and to assemble some related questions for improvement scholarship. We aim to highlight the centrality of aesthetics for both the ‘Q’ and the ‘I’ of QI—for both the evaluative and explanatory work that is needed to determine what ‘good healthcare’ is and to understand how and why we might succeed or fail to bring it about. In these two sections we will concentrate on the relevance of aesthetics to understanding the nature of quality but we will begin to indicate some implications for scholarly debate, and then turn to the theme of improvement scholarship more explicitly in the subsequent section. Our examples all combine both sensory and symbolic elements—a compound which we are treating as characteristic of aesthetics. We begin with those that have a strong material and sensuous dimension where it might be taken for granted that aesthetic considerations apply. After that we move on to examples which are equally sociomaterial but where we will put more emphasis on the arguably more neglected dimension that we are labelling as the ‘aesthetics of sociality’.

## The material dimension of healthcare—making and revising aesthetic discriminations

To indicate the non-marginal place of aesthetic considerations perhaps a good place to start is ‘medicine’—here, primarily referring directly to tablets, potions, infusions, creams or ointments; but, of course, ‘medicine’ is also a suggestive synecdoche. Medicines are a key interface between the healthcare system and patients and carers. When prescriptions are well judged medicines can do a significant proportion of the work of healthcare. They are also symbolically important, sometimes representing the trust and hope that people place in healthcare but also, some of the time, representing anxiety, dread and an authoritative stamp of ‘sickness’. However—stressing materiality for now—aesthetic considerations are at the heart of medicines' acceptability. This includes the shape and size of tablets, the taste, smell and viscosity of liquids, and the feel of, and sensations caused by, ointments. In turn acceptability is an important determinant of adherence (Liu *et al*. 2014). So aesthetic considerations, in the basic sense of the relative appeal or lack of appeal to various senses, are central to the design of pharmaceuticals and to supporting their use in practice (eg, help with splitting or crushing tablets, administering treatments and even ‘disguising’ the unpleasantness of certain medicines).

This familiar example provides an illustration of both the explanatory and evaluative importance of aesthetic considerations. It can also be used to show how aesthetic responses can both be constitutive of and a distraction from questions of technical effectiveness. A medicine that is relatively agreeable (as opposed to disagreeable) can be seen as better in that sense alone but may also be more effective for that same reason. The aesthetic reactions we have cited here are a part of what makes medicines work. They are one of the factors that can be used to explain non-adherence or altered to improve adherence. They are, in this regard, core to the technical effectiveness of medicines. Of course, someone could argue that it is really only the ‘active ingredients’ of medicines that determine their effectiveness and technical quality; but that line can be defended only on an implausibly narrow construction of what makes medicines ‘fit for purpose’. In the real world, especially in a world where healthcare is seen as about the interaction of biomedical and human factors, these aesthetic reactions cannot be pushed outside of conceptions of technical quality.

However, even though we should not always seek to drive a wedge between aesthetic and technical considerations a wariness about conflating them makes sense. There are cases where we might wish to question an appeal to aesthetics and problematise it as both superficial and potentially misleading. We can treat aesthetic acceptability as an important aspect of medicines’ quality in its own right, and as relevant to effectiveness, while also stressing that it clearly need not always coincide with what is best overall. This is something, for example, that critical consumers need to take into account when choosing over-the-counter (OTC) medicines. Pharmaceutical manufacturers and marketers are mindful of the aesthetic dimension of medicines and the need to respond to consumer perceptions and preferences. Work on acceptability in the OTC marketplace encompasses both sensory and symbolic appeal, characterising the ‘aesthetic attribute’ of medicines as:

the outward appearance of the product, feeling of comfort (ergonomics), utility, style, … as well as factors such as shape, dimensions, propositions, colour and finish of the product. The products’ aesthetic appeal can be built on emotional connection with the product. This can have a dramatic effect on patients’ compliance and can increase brand loyalty. (More and Srivastava 2010)

By raising the question of emotional appeal and by going on to discuss the role of aesthetics not only in the physical properties of drugs but in packaging, advertising and branding of products More and Srivastava effectively flag up the potential dangers of an elision between aesthetics and technical quality, because OTC medication is an area where it is well known that choices based on brand loyalty do not necessarily coincide with what is technically best.

This example raises the question of how we should frame the nature and boundaries of ‘aesthetic’ reactions and, in addition, whether there are means of discriminating between more and less well-grounded or meaningful aesthetic reactions and judgements. When, if ever, should we resist using aesthetic reactions as a guide to quality? We could, for example, simply describe anything that people say they ‘like’ or ‘feels good’ as an account of their aesthetic judgements. While this category of things is extremely important in explanatory accounts and must not be ignored, it also seems too ‘thin’ and open-ended to be sufficient on its own to play a major role in determining evaluative judgements about the quality of care. Indeed one of the criticisms sometimes made of the ‘everyday aesthetics’ literature is that is can operate with too elastic, inclusive and hence vague conception of the aesthetic, rendering the idea ‘contentless’ (eg, Forsey 2012, p203). However, as a counterweight to this, scholars working on everyday aesthetics (Irvin 2009; Leddy 2005) have argued that to treat something as an aesthetic judgement is to treat it as part of sociocultural life and, as such, to see it as a matter of ‘taste’ about which—while accepting that tastes are diverse—we can also deliberate and make and debate discriminations.

This discussion indicates that the suggestion made by Slater, McCance, and McCormack (2017) (cited above) that the physical environments of healthcare need to ‘balance aesthetics with function’ is worth underlining but also unpacking. There may be times when aesthetics and functionality can point in different directions and some ‘trade-off’ is necessary but there will also be times when the two are more closely integrated. More broadly, as indicated with the example of ‘green’ consumption or activism mentioned above, we can ask what scope there is to better integrate and align aesthetic, technical and ethical lenses so as to ensure, so far as possible, that our conceptions of each of these things informs the other, thereby enlarging and enriching our evaluative sensibilities. So, to be clear, in arguing that aesthetic considerations are of universal relevance in quality debates we are certainly not suggesting that they should always play an overriding role, nor even that aesthetic judgements should consistently feature prominently in all determinations of quality but rather that we should always be open to the possibility that they may emerge as having a very important, and sometimes decisive, role. Furthermore, as the case of medicines illustrates, it may be that we sometimes cannot properly identify and articulate technical or ethical dimensions of quality without attention to aesthetics.

The design and choice of prostheses is another area where aesthetics has conspicuous relevance. Of course, direct work on and with bodies, including links with ‘body image’, is the area of healthcare most associated with the word ‘aesthetic’. This usage sometimes refers, in a relatively restricted way, to people’s ‘looks’—to increasing a sense of attractiveness or, equally contested, to offering 'naturalness' or 'normalcy'. However, while once again aesthetics can be used to refer to something relatively superficial in this context it definitely need not. In the case of prosthetics for people with limb difference, for example, it is also deeply embedded with profound questions about identity, management of potential stigma, as well as ethical questions about how to understand and respectfully engage with the values, inclusion and agency of people with disabilities. It is also clearly poses aesthetic questions about a range of senses and feelings, and about self-expression and activity, not just ‘looks’ in a narrow sense.

Given this rich and demanding agenda the way that aesthetic questions are framed by those monitoring user experiences is very disappointing and typically neglects many of the serious questions that arise. For example, in the Prosthesis Evaluation Questionnaire (Boone and Coleman 2006; Legro *et al*. 1998) wearers are asked to rate ‘how your prosthesis has looked’ over the previous 4 weeks from ‘Terrible’ to ‘Excellent’; and whether the prosthesis has restricted choice of clothing, or damaged clothing. Clearly these concerns are significant but they do not go very far. There are a growing number of complex aesthetic possibilities in the field of prosthetics and these intersect with the identities of users in multiple ways (Pullin 2020). For example, a combination of technical and social developments has meant that the design of prosthetic hands has moved away from a paradigm in which (1) Aesthetics was largely equated with cosmetic adequacy and/or simulation of a ‘realistic look’, and hence (2) The demands of form and function—or aesthetic adequacy and technical adequacy—were often assumed to be in conflict. The design and choice landscape is now more contested. Some people, for example, may welcome high-tech robotic hands and see them as embodying a look and feel that they prefer (at least some of the time). And this may apply whether or not the technology is covered by silicone cosmetic gloves that are also available (in various skin tones). Importantly design and choice is always underdetermined by technological considerations, for example, a robotic hand need not reflect the art direction of science fiction films (Murray 2020). Not all people with limb difference want ‘realism’ and some are happy to embrace or play with making their differences overt in various ways including by invoking a ‘transhuman’ identity. By contrast others prefer more nuanced identity statements which neither deny nor ‘play up’ difference but seek to naturalise and normalise it. This context increases the importance, and some of the complexities, of shared decision-making in this area and highlights the centrality of aesthetic discussion and deliberation within it.

Interpretations of aesthetics which focus on the ‘surfaces’ of things rather than their underlying constitution and their associated personal and social meanings are obviously inadequate here. The danger of missing the ‘depth’ of objects also applies temporally. Our relationships with things evolve over time and our aesthetic discriminations also evolve and are shaped by these emergent relationships. This includes the way that the design of prostheses helps to shape the experiences and meanings of disability. These, and many other, designed objects can reflect or contradict, project or undermine, a range of different values and attitudes. They are very unlikely to be able to be neutral in this respect. Yet often the working assumption, overtly or implicitly, is that they are. The exhibition *Hands of X: Design Meets Disability* exhibition at the V&A Dundee (Pullin *et al*. 2019) explored this range of questions about prosthetics, identity and aesthetic discriminations including asking:

what might a prosthetic hand look like that was obviously artificial, unashamedly so, yet at the same time understated and *unremarkable*?

To further these debates the exhibition also explored the valuable lessons that might be learnt from the materials and craft traditions that informed the earlier development of prosthetics. This is an area where more can and should be done to explore the entanglement of aesthetic, technical and ethical questions. This includes: (1) Promoting greater reflexivity about the balances, and the possibilities of integration and alignment, between clinical-technical and aesthetic considerations; and (2) Ensuring future developments include, and are responsive to, a broad range of perspectives, including those coming from art and design and, of course most importantly, the diverse voices of people who have reason to make use of prostheses.

We have deliberately started from examples where material elements are prominent. But the example of prosthetics shows not only that, once we pay attention, our notion of what counts as aesthetic and aesthetically valuable can evolve, but also that this will likely include attention to personal and cultural meanings and to identities, biographies and self-expression. While we may start by thinking about discriminations in terms of what is more or less pleasant to the senses, we soon have to turn our attention to what, in Hume’s terms, gives more or less ‘satisfaction to the soul’ (Hume, Nidditch, and Lewis Amherst 1740, p299). Light and Smith 2005, p7) we do not need to (nor can we easily) separate out these two things. As noted in our initial account of everyday aesthetics, sensory and symbolic elements are entangled together and our aesthetic judgements respond to this combination.

We hope to have begun to illustrate how aesthetic reactions are often central to understanding what counts as success in healthcare such that our explanatory and evaluative frameworks are incomplete without them. If a healthcare system aspires to be ‘person-centred’ this automatically opens up aesthetic considerations. This follows from recognising that person-centredness includes, at a minimum, being responsive to ‘values and preferences’, and people navigate the world partly through their aesthetic values and preferences. Interacting sensory, affective and cultural experiences inform both unconscious motivations and the overt reasons people offer for choices and actions. Aesthetics is not an ‘add-on’ agenda for healthcare quality. Nor is it an agenda that can be embraced without some debate as to how it should be interpreted and applied both in contrast to, and in combination with, ethical and technical aspects of quality.

## The social dimension of healthcare—shaping and reshaping relationships

As we noted earlier one domain where aesthetic considerations already get some recognition within quality discourses is the physical environment. A focus on this domain makes sense and maintains the link with materiality—for example: the architecture of healthcare institutions; the division and organisation of space; furniture and furnishings; lighting; the overall configuration, arrangement and amenities attached to waiting; working and social areas; and so on. These are all topics with obvious aesthetic relevance. As with the objects discussed in the previous section they can all be understood as what service designers call ‘touchpoints’—points where users interact with services, which can be seen as somehow ‘speaking for’ the service as a whole. A thoughtful and poetic example is designer Kenya Hara’s signage for Umeda Hospital, Yamaguchi, Japan, in which printed white cotton sleeves are tied to signs (Cardenas 2016). That the fabric signs need to be frequently laundered and retied is a deliberate embodiment of the cleanliness of this maternity hospital as a whole. In a hospital, as in a range of settings, cleanliness indicates service, trust and security (Hara 1998).

However, healthcare environments are a compound of physical and social elements. As we all know physical environments can be designed to be welcoming, comfortable, peaceful, nurturing, and/or enlivening, exciting, and so on, but they will not succeed in being any of these things without people making them so. In this section we will place emphasis on the ‘social’ dimension of sociomaterial environments—on ‘social touchpoints’.

People entering a healthcare setting will encounter something like an ‘ambience’, ‘atmosphere’ or ‘climate’ that is both material and social (eg, see Julmi 2017; Wright 2019). The language here is self-consciously open and vague rather than definitive. As mentioned above one of the insights developed within everyday aesthetics scholarship is that there need be no clear-cut, single or stable entity, or obvious demarcating boundaries, to the ‘objects’ under investigation. Part of such a climate will be the result of the dispositions, comportment and ways of relating embodied in healthcare actors. Reactions to, and evaluations of, such climates and encounters have important aesthetic components that, once again, can be distinguished from (but intermingle with) technical and ethical considerations. Broadly speaking people can find their healthcare encounters more or less unpleasant, enjoyable, engaging, motivating or fulfilling. Obviously, reactions will also vary between people. In a policy context where person-centredness is valued then attending to such differences is key, because person-centredness overlaps with aspects of equity and inclusion. There is a need to be mindful that healthcare that feels welcoming and safe to some people may feel much less so to others (sometimes in a way that maps onto axes of difference such as class, gender, ethnicity or disability).

In addition to variations between people’s expectations, healthcare settings themselves play a range of very different functions. But some broad-brush generalisations about the aesthetic requirements of settings are possible. What counts as ‘fitting’ will reflect the role of the setting—some spaces may be intended to be more restful, cosy or even ‘homely’, others to be less warm but more functional or task-oriented, still others to be diverting, stimulating and conducive to activation, and so on. One of countless possible examples is psychotherapy spaces. Jackson (2018) characterises these spaces as typically combining home-like with office-like features designed to support the ‘holding environment’ that therapists seek to create by communicating ‘stability, comfort, and protection’. It is the role of the therapists and the space to offer a sanctuary or refuge. To work as a holding environment, in the sense derived from Winnicott (1953), the client must feel secure enough to confront painful subjects and to be able to contemplate the possibility of adaptation, and the therapist must also be able to be still and present and to feel that they are in an effective workplace. Not all care settings need to work as holding environments in this therapeutic sense but the idea can be applied more widely or extended by analogy. For example, it has been applied to therapeutic residential care for young people that combines accommodation and a garden space with roles and relationships designed to enable ‘emotional holding’ and ‘containment’ of difficult feelings (Vishnja 2007). Many other settings are partially comparable because patients can easily feel lost within, or threatened by, experiences of ill-health or by healthcare itself. The risks of feeling vulnerable, disengaged or disempowered are widespread and—as will go on to highlight—given the increasing aspirations towards co-production, this gives the aesthetics of sociality particular significance.

The crucial role of aesthetics in the area of professional comportment has long been recognised, for example, in discussions of etiquette and ‘bedside manner’. The Hippocratic Corpus itself includes recommendations on appearance and demeanour including:

Be solicitous in your approach to the patient, not with head thrown back (arrogantly) or hesitantly with lowered glance, but with head inclined slightly as the art demands (cited in Silverman 2012, p59).

Similarly, John Gregory’s advice on medical etiquette, written in the late eighteenth century warns about the risk of appearing too superior in dress or comportment, especially when dealing with certain patients including seriously unwell people or children:

the visit of a physician, even when wished for, is often particularly dreaded, as it naturally awakens the apprehensions of danger; apprehensions, which a formal dress, and a solemn behaviour, are ill calculated to dispel (Gregory 1772, 61; cited in Maio 1999).

Most recently, the rise of online consultations (fuelled substantially by the COVID-19 pandemic) has given rise to discussion of ‘webside manners’ that brings together approaches to physical-technical and social comportment. For example, the *Journal of Palliative Medicine* has published recommendations on ‘Maintaining Human Connection’ (Chua, Jackson, and Kamdar 2020) which includes advice on multiple factors including: body position and posture, best eye level, the structure and rhythm of conversation, and how to signal an emotional response, for example, ‘place hand over heart to convey empathy’ (p1508).

These examples illustrate the relevance of ‘negative aesthetics’ to healthcare quality—in these instances this means, for example, ways in which healthcare encounters may produce apprehension or alienation. There are obvious parallels with the discussion of medicines above. The factors that make settings and encounters aesthetically satisfactory or unsatisfactory will sometimes be constitutive of technical and ethical quality and may sometimes diverge. This may vary as we move along the spectrum between threshold notions of quality and ‘ideal’ notions. At the most negative end insufficient attention to aesthetic considerations may be an indicator that healthcare is poor. A setting that is inhospitable, shabby, unpleasant and possibly even unclean may be both unsafe and demotivating to both staff and patients. Here aesthetic, ethical and technical concerns can coincide—in circumstances where bad conditions are avoidable such a setting may not only produce needless risks but also signal too little care or respect. However, some aesthetic concerns may be of relatively superficial benefit or even become positively misleading. The latter connects to well-recognised worries about someone’s appealing bedside manners potentially concealing bad judgements and practices, or too much weight being attached to well-appointed spaces and not enough to whether the basic building blocks of effective care are also in place.

These examples also evoke a parallel with the successful ‘performance’ of roles and effective ‘staging’ in arts contexts such as theatre. There are good reasons to question how far such parallels are apt but there is no reason to entirely reject them. Of course, healthcare is not to be equated with art, and is oriented towards practical imperatives, but it is also a multisensory, multimodal set of cultural forms that rely on performances and dialogue, the construction and interpretation of narratives and metaphors, rhythm and improvisation, and so on (Geller 2018). Embracing this comparison opens up a potentially rich set of resources for talking about the aesthetics of healthcare encounters. For example, Maio (1999) suggests that attention to aesthetics is a way of opening up a consideration of a variety of forms and styles of communication that are underdetermined by ethics. As he notes there is an obligation to communicate with a patient before commencing the administration of chemotherapy, and this obligation determines some of the content of the communication, but it leaves open a range of styles. As with the case of prosthetics discussed above this allows for responsiveness to patients and for professionals to modulate, adapt and expand their approaches (assuming they do so within an authentic and broadly effective repertoire).

Bleakley, Marshall, and Brömer (2006) highlighted the possibility of viewing medical professionalism as a whole in aesthetic terms by placing an emphasis on ‘sensibility’ including, for example, ‘artistry’ and ‘connoissuership’ in knowledge and practice. They have also demonstrated the value of directly ‘translating’ aesthetic categories into healthcare practice by, for example, showing the central relevance of a Homerian ‘lyrical aesthetic’ to the achievement of a form of professional practice that is empathic and does not overemphasise cure at the expense of person-centred care (Bleakley and Marshall 2012)—the very notion of 'cure', of course, being problematic in many instances including in the area of disability. This is one reminder that another fundamental way in which settings and encounters can ‘fail’ aesthetically, and with substantial practical consequences, is that—however professionally maintained and fronted they are—they may be experienced as barren, inert or ‘cold’. This will also amount to a critical technical and ethical failure in cases where engagement, connection and partnership working are seen as crucial facets of care (and of course these ambitions are increasingly core features of quality discourses).

It is noteworthy that the recent high-profile critique of the industrialisation of care by Allwood *et al*. (2021) echoes some of the language cited above about rhythm and the aesthetics of sociality by arguing for what the authors call ‘elegance’ in communication. In contrast to ‘hurried’ conversations—motivated by efficiency but actually inefficient—elegance, the authors argue, may not require more time and can also save much waste of time and other resources elsewhere. Rather, ‘Elegance involves protecting the ability of patients and clinicians to set the tempo of their interaction, a tempo that encourages noticing what is the matter and responding in a way that reflects what matters’ (2021, p2). In this context attention to work on the aesthetics of conversations might provide some helpful clues. For example, Puolakka (drawing on both Dewey and Davidson) analyzes aesthetically successful conversations as those in which meaning is conserved, grows and is accumulated, in which there is genuine mutual engagement, the exercise of empathy and imagination, the possibility of absorption and an internal momentum that, ideally, moves towards a consummation rather than a mere cessation (Kalle 2017). Anyone with experience of healthcare encounters will recognise the relevance of this to their experiences. Even when—arguably especially when—the substantive ground is practically and emotionally challenging, our encounters can vary hugely in terms of how satisfactory we judge them to be through this aesthetic lens.

Allwood *et al*’s critique is motivated by a conception of person-centredness that stresses the primacy of kindness and compassion, but which also hopes to shift the centre of gravity towards the co-production of care. Aspirations for co-production arguably reframe what count as ‘negative aesthetics’. As the comportment examples above indicate, power is mediated aesthetically—and of course this is also well known from extreme cases such as the work of Canetti (1962) illuminating the aesthetics of Fascism—and so successful efforts to create environments and encounters that support co-production will depend on considerable investment of thought and discussion into aesthetic reimagining. For a space to support co-production it will not be enough for it to be non-silencing, or even to feel welcoming, but it will need to help facilitate power-sharing. In crude terms we might suggest that for healthcare encounters to support co-production we need to know how to shift them along a spectrum from cold to warm to ‘very warm’.

One way to investigate the aesthetics of sociality in healthcare is to draw on experience of arts-based interventions. These cover an immensely wide range of possible forms of enrichment and styles of working against ‘aesthetic deprivation’ (Berleant 2010; Moss and Desmond 2014). Arts-based working is advocated as contributing directly to health and well-being, as enabling inclusion and a degree of tailoring to cultural difference and as fostering self-expression and creativity (Daisy and Finn 2019; Gardner *et al*. 2021). We will just mention one indicative example, namely Guddi Singh’s account (RAD 2021) of the introduction of regular dancing sessions—involving both patients and staff—into paediatric wards. This account points towards the dramatic difference such an initiative can make—in terms of breaking down barriers, encouraging confidence, mobilising energy, renewing relationships, building community, strengthening staff well-being and teamwork, and not least in terms of simple joy. One important way of taking this kind of intervention seriously is, obviously, to advocate for more arts-based working in quality improvement. But, alongside that, we should look for some broadly analogous ways for us reimagine and extend routine (non-arts-based) working practices.

## Extending improvement scholarship

We are arguing that being ready to talk about everyday aesthetics provides an important additional resource for QI. This, we propose, entails improvement-related scholarship also embracing aesthetics so it is better able to support these discussions on the ground. Many people working in healthcare improvement are already interested in aesthetic factors but relatively few talk openly in these terms. Nor are aesthetic aspects of working often consolidated together or treated as a professionally or institutionally recognised dimension of quality. Putting everyday aesthetics on the map may not be easy because, as signalled earlier, for some people the very idea of aesthetics may be off-putting—perhaps because it seems to refer to something superficial or, on the other hand, to something esoteric and obscure. Hopefully we have said enough to underline the clear relevance of aesthetics and to show that embracing this relevance entails some explicit attention to potential complexities and tensions. We see a potential parallel here with the growth of ethics discussion in healthcare settings including in QI. Ethics is still seen as an area where some scholarly fields and communities have particular expertise but, at the same time, it has increasingly become a normalised part of discourse such that all kinds of healthcare actors feel comfortable about raising and debating everyday ethical issues. Of course things can go wrong in this process—for example, people may sometimes operate with overly simplified—perhaps unhelpfully rigid or loose—conceptions of ethics (or here aesthetics). This should make us cautious about *how* aesthestics is incorporated into QI. In particular we should be very wary of anything like a ‘checklist’ approach to aesthetics that indirectly encourages would-be improvers to quickly identify and attach weight to specific aesthetic judgements. As we have seen aesthetic considerations need to be sifted, filtered and carefully weighed. For the same reasons the incorporation of aesthetics into QI courses, or medical education more broadly, should centre on the cultivation of imagination and critical debate rather than on a rush towards practical operationalisation. But some such incorporation still seems to us a substantial improvement over silence or complete deference to the ‘experts’.

In terms of improvement scholarship Experience Based Co-Design (EBCD) provides a strong platform for others to build around. EBCD is a well-established current within the broad family of healthcare quality improvement that takes as its starting point the way people feel about their healthcare experiences (as users and providers)—explicitly underpinned by the language of aesthetics as a core component of design; for example, it applies the heuristic of ‘touchpoints’ from design aesthetics (Donetto *et al*. 2015). It is also directed towards improvement as a co-productive process. Our thought is that there is considerable scope for extending and ‘normalising’ such conversations about everyday aesthetics across the many others (beyond those working in EBCD) interested in the theory and practice of quality improvement and, in so doing, also to enhance the breadth and depth of conversations and debates by welcoming many voices from academic aesthetics and design, arts-based working, as well as those of patients, families and professionals, into QI more broadly.

Our suggestion simply encourages processes that are already underway. The knowledge base of QI has broadened in recent years such that ‘improvement science’ is increasingly extending and becoming ‘improvement scholarship’ (Cribb 2018). This is partly because the heavily technicist and psychological roots of QI in the industrial sector from which it sprang have needed to be complemented to make it suited for the healthcare sector, especially as models of healthcare provision, and conceptions of healthcare purposes and processes, have broadened out. But it is also a product of the raised profile and growth of the QI sector which has attracted interest in, and involvement from, social movements and scholars from the social sciences such as sociology who have often added critical as well as problem-solving perspectives to the interdisciplinary mix (eg, Allen *et al*. 2016). Ethics and medical humanities (eg, Boulton, Sandall, and Sevdalis 2020; Cribb, Entwistle, and Mitchell 2020; Palmer *et al*. 2019) are now also seen as potentially valuable contributors to what is becoming a more diverse and liberal field and given this trajectory it makes sense for healthcare aesthetics to find a more prominent place at the table.

Our core argument is threefold: (1) Deciding what counts as ‘good’ healthcare has to encompass debate about aesthetic as well as ethical and technical considerations; (2) Aesthetic considerations can play a critical explanatory as well an evaluative role and therefore can inform approaches to improvement; and (3) As QI is increasingly embracing broader paradigms, including co-production models, an aesthetics lens may now prove especially valuable. We have touched on these points in passing but we will briefly summarise them here and, following that, we will also pull together some of the questions our account raises for debate within both practice and scholarship.

Of course, the central case for the relevance of aesthetics to QI is that aesthetic quality is an important dimension of healthcare quality. And unless would-be improvers have an informed and relatively broad picture of the range of things that constitute success, they cannot go about planning their improvement activities in a defensible way. As we have also tried to illustrate, aesthetic factors are not an overriding consideration. Sometimes they may play a key constitutive role in supporting technical and/or ethical quality, even at quite a basic level, but sometimes they may pull against these concerns.

We have also tried to indicate that aesthetics can make a crucial contribution to explanations and thereby to steering possible change. There can be aesthetic reasons for action (or inaction) (Lesser 1972) and aesthetic reactions may also underpin unconscious motivations. Unless we are aware of this explanatory role our ‘theories of change’ will sometimes be incomplete or even mistaken. In a crude way this is captured in the example of the influence of sensory responses on medicines adherence but it applies far more broadly. If the sensory and symbolic textures of healthcare make people experience and envisage healthcare as threatening or cold then this will not only make them slow to come forward, but may also make them feel unseen, unheard and uncared for when they do, resulting in fundamental failures in both effectiveness and person-centredness. Equally these features can make professionals feel disconnected from their workplaces and alienated from their vocational identities.

Returning to person-centredness takes us on to the reason the kinds of aesthetic considerations we have discussed are arguably particularly salient in this phase of the development of QI and improvement scholarship. Mary Dixon Woods has written about the need for QI to move ‘beyond effectiveness’ (Dixon-Woods 2019). This signals that it is not just the knowledge base but also the substantive agenda and ‘logics’ of improvement practice that needs to expand—of course clinical effectiveness matters but other things also matter and it is important for QI to reflect on the full range of values and norms that inform it and that it serves. This, as Dixon-Woods makes clear, includes attention to themes such as power and inequality and includes responding to the calls for the co-production of healthcare and healthcare improvement. Related work on QI has also stressed the importance of ‘context-strengthening’ approaches to QI as well as approaches based on specific interventions (noting that contexts and interventions are co-constitutive) (Liberati *et al*. 2019). In their work on ‘very safe maternity care’, for example, Liberati *et al* identify—among other things—important factors contributing to success that might be thought of as ‘soft’ aspects of healthcare sociality including collegiality, mutual respect, open discussion, organisational citizenship, attention to ‘soft intelligence’ not just hard data, and space for staff to mix socially and professionally.

It would be reasonable, at this point, for someone to argue that we could accept the central relevance of aesthetic considerations but still avoid the potentially off-putting language. That practical discussions and debates about aesthetics could be had in terms of what people find ‘satisfying’ or ‘appealing’ and so on. This sounds plausible but only up to a point. First, it seems much would be gained by opening up richer, more differentiated, aesthetic vocabularies whether by drawing on academic aesthetic discussions or qualitative research traditions (such as in EBCD). Second, we would, again, invoke the parallel with ethics and suggest that there are important uncertainties and tensions raised by the inclusion of aesthetic considerations in QI that it is valuable to ‘name’ and debate. There is the question of when we should be suspicious of appeals to aesthetics—when it may send us in directions that are potentially damaging. The discussion of potential tensions between aesthetic, ethical and technical dimensions of quality can contribute to an understanding of improvement goals in at least two respects. It provides another reminder that there may be trade-offs between rival conceptions of good healthcare that we need to navigate through. And it also poses valuable meta-questions about how far we can or should shape or ‘educate’ our sensibilities so that there is an optimum degree of alignment between and integration of our aesthetic judgements and those that relate to other dimensions of quality. Drawing on aesthetic considerations to plan or mould improvements is not easy. It throws up dilemmas of its own. These include dilemmas about how far to try to accommodate differences in aesthetic reactions and sensibilities (eg, to respond to cultural differences), and how to think about and manage such balances in settings where both public and more personalised activities and ‘spaces’ come together.

Emerging emphases and paradigms in QI pose some exceptionally difficult challenges. They involve trying to forge new roles and relationships including much more meaningful forms of partnership working between staff and across professional patient and organisational boundaries (Cribb and Collins 2021). On older paradigms what mattered was arguably that healthcare settings were not too aversive but sufficiently non-threatening to enable professionals to engage effectively with patients. Newer paradigms call for spaces and encounters that positively welcome, inspire and help mobilise the interest, self-expression and agency of patients and which foster ongoing mutual understanding and co-creation. If we want to improve healthcare by making it more inclusive and co-productive we are unlikely to do that without being attentive and sensitive to what healthcare environments and encounters express, communicate and open up for discussion. It is no coincidence that a movement such as EBCD that is organised around co-production asks these questions and acknowledges the importance of aesthetics. The challenge is to translate the metaphors used above about the need for more ‘dancing’ in healthcare into a revitalised and enlarged ‘choreography of relationships’.

## Conclusion

What we are proposing is perhaps nothing much more than a trick of perspective. It is a reframing of healthcare improvement which involves something like switching around figure and ground. Normally it seems that the technical-functional aspects of QI are placed in the forefront and that ethical and to some extent aesthetic considerations are included, but make up the background. We are raising the question about how the way we see, and our approach towards, technical-functional questions might evolve if we spent some of the time placing aesthetics in the foreground.

The language, and the growing current of work on everyday aesthetics, helps to mainstream aesthetics as both a routine and pervasive feature of our lives, including our professional and institutional lives. As we have tried to indicate it allows us to use an aesthetics lens to think not only about a subset of relevant ‘designed objects’ but also about the more diffuse and sometimes inchoate ‘objects’, like ‘climates’, that we create without any discernible design or designer. In so doing we can pursue many of the agendas that are associated with arts-based working but apply them to a much more extensive and mundane territory. For example, we can see how the distinction between ‘presentational’ and ‘participatory’ arts-based working (eg, Cao, Blinderman, and Cross 2021) has implications for both the material and social dimensions of everyday aesthetics. This distinction is mirrored, for example, in each of the two discussions above—of medicines and prostheses, and of ‘bed-side manners’ and co-production—and is indicative of the potential for everyday aesthetics to help support the shift from ‘consumption’ to ‘co-creation’. Indeed, we have suggested, it is this shift of emphasis within healthcare and QI discourses that make now a good time to more wholeheartedly embrace, and make use of, the theoretical and practical resources of aesthetics. Aesthetics is an everyday matter and talking about it should, we think, become more of an everyday norm in healthcare improvement.

## Data Availability

No data are available.

## References

[R1] Allen D. , Braithwaite J. , Sandall J. , and Waring J. , eds. 2016. The Sociology of Healthcare Safety and Quality. Chichester: Wiley Blackwell. 10.1002/9781119276371. 26679563

[R2] Allwood D. , Koka S. , Armbruster R. , and Montori V. . 2021. “Leadership for Careful and Kind Care.” BMJ Leader. 10.1136/leader-2021-000451. PMC12038097 36170535

[R3] Bate P. , and Robert G. . 2007. Bringing User Experience to Healthcare Improvement: The Concepts, Methods and Practices of Experience-Based Design. Abingdon: Radcliffe Publishing Ltd.

[R4] Berleant A . 2010. Sensibility and Sense: The Aesthetic Transformation of the Human World. Exeter: Imprint Academic.

[R6] Bleakley A. , Marshall R. , and Brömer R. . 2006. “Toward an Aesthetic Medicine: Developing a Core Medical Humanities Undergraduate Curriculum.” The Journal of Medical Humanities 27 (4): 197–213. 10.1007/s10912-006-9018-5. 17096192

[R5] Bleakley A. , and Marshall R. J. . 2012. “The Embodiment of Lyricism in Medicine and Homer.” Medical Humanities 38 (1): 50–54. 10.1136/medhum-2011-010138. 22792556

[R7] Boone D. A. , and Coleman K. L. . 2006. “Use of the Prosthesis Evaluation Questionnaire (PEQ).” JPO Journal of Prosthetics and Orthotics 18 (Proceedings): P68–79. 10.1097/00008526-200601001-00008.

[R8] Boulton R. , Sandall J. , and Sevdalis N. . 2020. “The Cultural Politics of ‘Implementation Science.’” The Journal of Medical Humanities 41 (3): 379–94. 10.1007/s10912-020-09607-9. 31965463 PMC7343725

[R9] Budd M . 2008. Aesthetic Essays. Oxford: Oxford University Press. 10.1093/acprof:oso/9780199556175.001.0001.

[R10] Canetti E . 1962. Crowds and Power. New York: The Viking Press.

[R11] Cao E. L. , Blinderman C. D. , and Cross I. . 2021. “Reconsidering Empathy: An Interpersonal Approach and Participatory Arts in the Medical Humanities.” The Journal of Medical Humanities 42 (4): 627–40. 10.1007/s10912-021-09701-6. 34100177 PMC8664795

[R12] Cardenas D . 2016. “Umeda Hospital / Kengo Kuma & Associates.” ArchDaily. https://www.archdaily.com/792313/umeda-hospital-kengo-kuma-and-associates.

[R13] Carroll N . 2001. Beyond Aesthetics. Cambridge: Cambridge University Press. 10.1017/CBO9780511605970.

[R14] Chua I. S. , Jackson V. , and Kamdar M. . 2020. “Webside Manner during the COVID-19 Pandemic: Maintaining Human Connection during Virtual Visits.” Journal of Palliative Medicine 23 (11): 1509: 1507–9. 10.1089/jpm.2020.0298. 32525744

[R17] Cribb A . 2018. “Improvement Science Meets Improvement Scholarship: Reframing Research for Better Healthcare.” Health Care Analysis 26 (2): 109–23. 10.1007/s10728-017-0354-6. 29270810 PMC5899995

[R15] Cribb A. , and Collins A. . 2021. “Strengthening Citizenship: A Healthcare Improvement Priority.” Future Healthcare Journal 8 (1): e174–77. 10.7861/fhj.2020-0122. 33791503 PMC8004299

[R16] Cribb A. , Entwistle V. , and Mitchell P. . 2020. “What Does ‘quality’ Add? Towards an Ethics of Healthcare Improvement.” Journal of Medical Ethics 46 (2): 118–22. 10.1136/medethics-2019-105635. 31732680 PMC7035683

[R22] Daisy F. , and Finn S. . 2019. What Is the Evidence on the Role of the Arts in Improving Health and Well-Being? A Scoping Review. Copenhagen: WHO Regional Office for Europe. 32091683

[R18] Dewey J . 1958. Art as Experience. New York: Capricorn Press.

[R19] Dixon-Woods M . 2019. “How to Improve Healthcare Improvement.” BMJ (Clinical Research Ed) 367: l5514. 10.1136/bmj.l5514. PMC6768008 31575526

[R20] Donabedian A . 1989. “Institutional and professional responsibilities in quality assurance”. International Journal for Quality in Health Care 1 (1): 3–11. 10.1093/intqhc/1.1.3. 2490950

[R21] Donetto S. , Pierri P. , Tsianakas V. , and Robert G. . 2015. “Experience-Based Co-Design and Healthcare Improvement: Realizing Participatory Design in the Public Sector.” The Design Journal 18 (2): 227–48. 10.2752/175630615X14212498964312.

[R23] Forsey J . 2012. The Aesthetics of Design. Oxford: Oxford University Press.

[R24] Gardner H. , Leeding J. , Stanley E. , Ball S. , Leach B. , Bousfield J. , Smith P. , and Marjanovic S. . 2021. Arts-Based Engagement with Research. Cambridge: The Healthcare Improvement Studies Institute, University of Cambridge.

[R56] Geller J . 2018. “The Transformative Powers of Aesthetic Experiences in Psychotherapy.” Journal of Clinical Psychology 74 (2): 200–207. 29315547 10.1002/jclp.22582

[R37] Gregory J . 1772. Lectures on the Duties and Qualifications of a Physician. London: Strahan and Cadell.

[R25] Hara K . 1998. “Umeda Hospital.” https://www.ndc.co.jp/hara/en/works/2014/08/umedahospital.html.

[R26] Hume D. , Nidditch P. H. , and Lewis Amherst S.-B. . 1740. David Hume: A Treatise of Human Nature (Second Edition). Oxford: Oxford University Press. 10.1093/actrade/9780198245872.book.1.

[R27] Irvin S . 2009. “Aesthetics of the Everyday.” In A Companion to Aesthetics, edited by Higgins Kathleen , Hopkins Robert , Stecker Robert , and Cooper David . Chichester: John Wiley and Sons Ltd.

[R28] Jackson D . 2018. “Aesthetics and the Psychotherapist’s Office.” Journal of Clinical Psychology 74 (2): 233–38. 10.1002/jclp.22576. 29318597

[R29] Julmi C . 2017. “The Concept of Atmosphere in Management and Organization Studies.” Organizational Aesthetics 6 (1): 4–30.

[R46] Kalle Puolakka . 2017. “The Aesthetics of Conversation: Dewey and Davidson.” Contemporary Aesthetics 15 (1). https://contempaesthetics.org/newvolume/pages/article.php?articleID=791.

[R30] Leddy T . 2005. “The Nature of Everyday Aesthetics.” In The Aesthetics of Everyday Life, edited by Light Andrew and Smith Jonathan M. . New York: Columbia University Press.

[R31] Legro M. W. , Reiber G. D. , Smith D. G. , del Aguila M. , Larsen J. , and Boone D. . 1998. “Prosthesis Evaluation Questionnaire for Persons with Lower Limb Amputations: Assessing Prosthesis-Related Quality of Life.” Archives of Physical Medicine and Rehabilitation 79 (8): 931–38. 10.1016/s0003-9993(98)90090-9. 9710165

[R32] Lesser A. H . 1972. “Aesthetic Reasons for Acting.” The Philosophical Quarterly 22 (86): 19. 10.2307/2218588.

[R33] Liberati E. G. , Tarrant C. , Willars J. , Draycott T. , Winter C. , Chew S. , Dixon-Woods M. , et al . 2019. “How to Be a Very Safe Maternity Unit: An Ethnographic Study.” Social Science & Medicine (1982) 223: S0277-9536(19)30033-4: 64–72. 10.1016/j.socscimed.2019.01.035. PMC6391593 30710763

[R34] Light A. , and Smith J. M. , eds. 2005. The Aesthetics of Everyday Life. New York: Columbia University Press.

[R35] Liu F. , Ranmal S. , Batchelor H. K. , Orlu-Gul M. , Ernest T. B. , Thomas I. W. , Flanagan T. , and Tuleu C. . 2014. “Patient-Centred Pharmaceutical Design to Improve Acceptability of Medicines: Similarities and Differences in Paediatric and Geriatric Populations.” Drugs 74 (16): 1871–89. 10.1007/s40265-014-0297-2. 25274536 PMC4210646

[R36] Maio G . 1999. “Is Etiquette Relevant to Medical Ethics? Ethics and Aesthetics in the Works of John Gregory (1724-1773).” Medicine, Health Care, and Philosophy 2 (2): 181–87. 10.1023/a:1009901419932. 11080984

[R38] More A. , and Srivastava R. . 2010. “Aesthetics in Pharmaceutical OTC Marketing.” SIES Journal of Management 7 (1): 65–96.

[R39] Moss H. , and Desmond O. . 2014. “Aesthetic Deprivation in Clinical Settings.” Lancet (London, England) 383 (9922): 1032–33. 10.1016/s0140-6736(14)60507-9. 24665475

[R40] Murray S . 2020. “Disability, Technology and the Stuff of Science Fiction’. Imagining Technologies for Disability Futures Website.” https://itdfproject.org/disability-technology-and-the-stuff-of-science-fiction/.

[R41] Naukkarinen O . 2017. “Everyday Aesthetics and Everyday Behavior.” Contemporary Aesthetics 15.

[R42] Palmer V. J. , Weavell W. , Callander R. , Piper D. , Richard L. , Maher L. , Boyd H. , et al . 2019. “The Participatory Zeitgeist: An Explanatory Theoretical Model of Change in an Era of Coproduction and Codesign in Healthcare Improvement.” Medical Humanities 45 (3): 247–57. 10.1136/medhum-2017-011398. 29954854 PMC6818522

[R43] Parsons G . 2016. The Philosophy of Design. Cambridge: Polity Press.

[R44] Pullin G . 2020. “Ottobock Bebionic Hand.” Domus 1046 (May 2020): 50–55.

[R45] Pullin G. , Cook A. , More M. , Bassam L. , Clark B. , Gannon A. , and McMullan C. . 2019. “Hands of X: Design Meets Disability.” V&A Museum of Design Dundee, 27 Jun-1 Sep 2019. https://www.vam.ac.uk/dundee/exhibitions/hands-of-x.

[R50] RAD . 2021. Why Dance Matters, Episode 6. London: Royal Academy of Dance. https://www.listennotes.com/podcasts/why-dance-matters/episode-6-dr-guddi-singh-FYkRePuMwOI/.

[R47] Saito Y . 2007. Everyday Aesthetics. Oxford: Oxford University Press. 10.1093/acprof:oso/9780199278350.001.0001.

[R48] Saito Y . 2017. Aesthetics of the Familiar. Everyday Life and World-Making. Oxford: Oxford University Press.

[R49] Silverman B. D . 2012. “Physician Behavior and Bedside Manners: The Influence of William Osler and The Johns Hopkins School of Medicine.” Proceedings (Baylor University. Medical Center) 25 (1): 58–61. 10.1080/08998280.2012.11928784. 22275787 PMC3246857

[R51] Slater P. , McCance T. , and McCormack B. . 2017. “The Development and Testing of the Person-Centred Practice Inventory - Staff (PCPI-S).” International Journal for Quality in Health Care 29 (4): 541–47. 10.1093/intqhc/mzx066. 28586441

[R52] Vishnja A . 2007. “The Concept of the Therapeutic Holding Environment. Goodenoughcaring Journal 22 May.” https://goodenoughcaring.com/the-journal/the-concept-of-the-therapeutic-holding-environment-and-how-it-has-been-implemented-in-the-residential-home-for-young-people-in-which-i-work/.

[R53] Winnicott D . 1953. “Transitional Objects and Transitional Phenomena.” International Journal of Psychoanalysis 34: 89–97. 13061115

[R54] Wright S . 2019. “From ‘holding Pen’ to ‘a Space to Breathe’: Affective Landscapes in a Newly-Integrated Sexual Health Clinic.” Sociology of Health & Illness 41 (4): 806–20. 10.1111/1467-9566.12852. 30714162

[R55] Yanagi S . 1926. The Beauty of Miscellaneous Things. Reprinted in The Beauty of Everyday Things. 2018. London: Penguin.

